# Improvement of the Weather Resistance of a Selective Laser-Sintered Copolyester–Limestone Composite Using UV-326 and UV-328

**DOI:** 10.3390/polym12092079

**Published:** 2020-09-13

**Authors:** Huu Chinh Nguyen, Yanling Guo, Tat Thang Nguyen

**Affiliations:** 1 College of Mechanical and Electrical Engineering, Northeast Forestry University, Harbin 150040, China; chinhckcdn@gmail.com; 2 Phu Tho College of Technology and Agriculture, Phu Tho 35910, Viet Nam; 3 College of Wood Industry and Interior Design, Vietnam National University of Forestry, Hanoi 156200, Vietnam; thangnt@vnuf.edu.vn

**Keywords:** weather resistance, copolyester–limestone composite, UV-326, UV-328

## Abstract

A copolyester–limestone composite fabricated with selective laser sintering technology is a potential material for the repair of ancient brick structures damaged by the sun and rain, however the weather resistance of this material must be improved. Herein, UV-236 and UV-328 were employed as UV stabilizers and added into the composite. The results show that the addition of UV-326 and UV-328 effectively inhibited the degradation of CH and ester groups and the formation of hydroxyl, carbonyl, and carboxyl groups. Thus, the stabilizers significantly reduced the color change and decline in mechanical properties of the composite under sun and rain conditions. The proposed strategy can be used for the repair of damaged precious brick buildings.

## 1. Introduction

Ancient structures built with stones and bricks are precious treasures. However, the original colors and shapes of these structures can be lost due to severe damage by sun and rain over a long time [1]. Therefore, there is an urgent need for repairs and maintenance of ancient buildings. However, there are several challenges in using similar feedstock, such as limestone (main component: CaCO_3_), to prepare the parts for the repair of these buildings with traditional methods due to their complex surface structures and patterns. Since it was pioneered in the 1980s, three-dimensional printing (3D printing) as an additive manufacturing technology has been widely expected to revolutionize the rapid manufacturing of complicated parts [2]. Selective laser sintering (SLS) is a 3D printing technology that uses 3D data to prepare scaffolds by sequentially sintering the powder using lasers [3]. Among the frequently used 3D printing technologies, SLS has unparalleled advantages as it is applicable for a wide range of materials, no support is required during machining, and material that is not sintered can be recycled and reused. Additionally, the parts manufactured by SLS have high precision and mechanical strength [4]. Hence, SLS technology is promising for the maintenance of ancient buildings. However, it is challenging to use limestone as a raw material for SLS technology because limestone cannot be directly sintered by lasers due to its ultra-high melting temperature (the melting temperature of CaCO_3_ is above 1300 °C).

There is a pressing need for a suitable material that can easily be melted so that it can bind limestone particles together during sintering. As an excellent thermoplastic polymer, copolyester has been proven to be a suitable binder with good laser processing properties. Additionally, copolyester has been widely used in multiple fields owing to its superb heat resistance, physical and mechanical properties, and insulation properties. Especially, it can be used continuously at high temperatures (about 200 °C) and remains stable during rapid temperature changes [5]. Therefore, a copolyester–limestone composite prepared using limestone particles as the main component and copolyester as the binder and with the use of SLS technology is promising for use as replaceable parts in ancient buildings.

**Figure 1:**
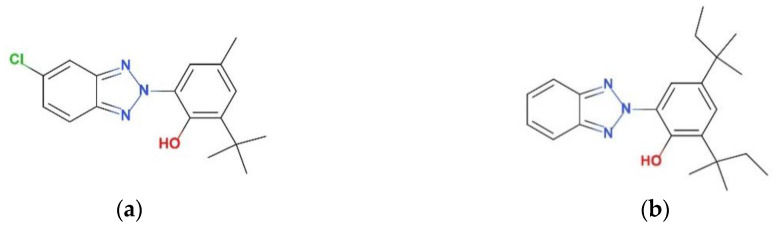
Chemical structures of (**a**) UV-326 and (**b**) UV-328.

Nevertheless, attention must be paid to the anti-aging properties of the SLS parts made with copolyester–limestone composites, as they are exposed to outdoor surroundings. The ancient structures made with the same raw materials are losing their original colors and shapes due to sun and rain damage and gradual aging [6]. UV-326 and UV-328 are two common UV stabilizers, which in general are used to improve the anti-aging performance of materials (The chemical structures of UV-326 and UV-328 are shown in Figure 1). They are potential additives that could increase the weather resistance of SLS parts [7]. Therefore, the objective of this work is to reduce the negative impacts on the color and mechanical properties of SLS parts brought about by sun and rain damage by employing UV-326 and UV-328, and to further understand the enhancement mechanisms of the anti-aging properties.

## 2. Materials and Methods

### 2.1. Materials

Limestone powder and copolyester flour were both obtained from Shanghai Tian Nian Materials Technology Ltd. (Shanghai, China). The diameters of most limestone and copolyester particles ranged from 43 to 55 μm. The main functional groups of the copolyester are ester and hydroxyl groups without aromatic groups. The limestone powder was dried for 8 h at 55 °C before mixing with copolyester. UV-326 (98% purity, melting point ranged 140–141 °C) and UV-328 (98% purity, melting point ranged 80–88 °C) were supplied by J&K Chemicals (Guangzhou, China).

### 2.2. Preparation of Copolyester/Limestone Composite

Limestone powder and copolyester flour were mixed at a ratio of 1:3 along with 3 wt% UV-326 or UV-328. The mixing was performed using an SHR50A high-speed mixer (Zhangjiagang Hongji Machinery Ltd., Dongying, China) for 15 min below 30 °C at a low speed of 750 rpm, then for 5 min at a high speed of 1500 rpm. Then, the mixed powder was removed from the high-speed mixer and naturally cooled to obtain powder for SLS.

**Figure 2:**
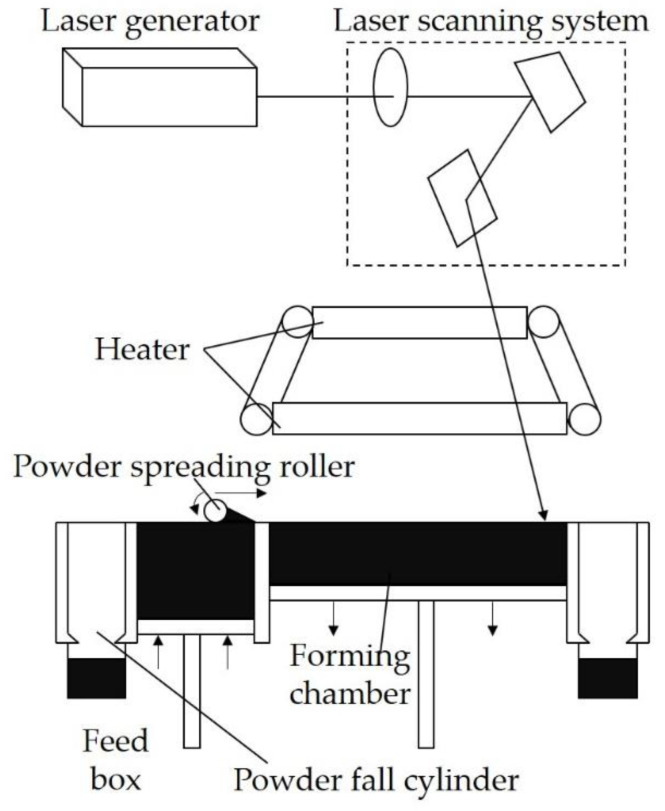
Schematic diagram of the SLS process.

The sintering process for 3D parts was conducted on an AFS-360 rapid prototyping instrument (Beijing Longyuan Technology Ltd., Beijing, China) equipped with a CO_2_ laser generator (wavelength of 10.6 μm and laser power of 55 W), laser scanning system, heat control system, powder spreading system, and working cylinder. The 3D parts were prepared using a computer-aided design model by repeatedly depositing ultra-thin layers of fusible powder and selectively sintering each layer onto the next layer with a modulated laser beam. Specifically, the fusible powder was spread by a powder-spreading roller on the top surface of the forming chamber, then melted under the laser beam repeatedly and combined with the previous layer. The 3D parts fabricated with copolyester, limestone, and UV-326 were designated as P/L-6, while those made with UV-328 were designated as P/L-8. The control group was prepared in the same way but without the addition of UV stabilizer, which was designated as P/L. The sintering process is presented in Figure 2 and the main process parameters are listed in Table 1.

### 2.3. Weather Resistance Examination

The weather resistance examination was conducted through an accelerated aging test performed on a Q-panel QUV aging tester (Q-Lab Corporation, Westlake, OH, USA) equipped with UVA-340 lamps. The tester can reproduce the damage that may occur to buildings after months or years of outdoor exposure. Each 12-h accelerated aging cycle included exposure to ultraviolet light at 60 °C for 8 h, followed by water spraying at 50 °C for 4 h. The UV irradiance at 340 nm was 0.89 W/m^2^. The materials were evaluated after accelerated aging exposures for 0, 5, 10, 15, 20, 25, and 30 d.

### 2.4. Color Measurement

The colors of samples were measured after accelerated aging exposures for 0, 5, 10, 15, 20, 25, and 30 d. At each period of exposure to accelerated aging, two samples were used for color measurements, and three measurements were performed for each sample.

The colors of SLS parts were measured using a CM-2300d spectrophotometer (D5003908, Konica Minolta Sensing, Inc., Tokyo, Japan) on a holder with a diameter of 8 mm. The overall color changes ΔE* were measured using the CIE 1976 L*a*b* color measuring system, and ΔE* values were calculated according to the following formulas:

(1) $$\Delta L^{*}=L^{*1}-L^{*0}$$

(2) $$\Delta a^{*}=a^{*1}-a^{*0}$$

(3) $$\Delta b^{*}=b^{*1}-b^{*0}$$

(4) $$\Delta E^{*}=\sqrt{\Delta L^{*2}+\Delta a^{*2}+\Delta b^{*2}}$$

where superscript *^1^ and *^0^ refer to values before and after exposure to accelerated aging; ΔL*, Δa*, and Δb* are the color changes before and after exposure to accelerated aging [8].

### 2.5. Mechanical Properties

The mechanical properties of SLS parts were tested using a CMT5504 tensile testing machine (TMS System Co., Ltd. Shenzhen, China) and a TCJ-4 impact testing machine (Tai He Testing Machine Limited, Jilin Province, China). The test protocol was as follows. The tensile strength was measured according to the ISO527-2 standard, with a crosshead speed of 5 mm/min and gauge length of 50 mm. The three-point bending strength was determined according to ISO178 standard at a crosshead speed of 5 mm/min and span of 64 mm. The U-shaped notch impact strength was determined as per ISO179-2 standard, with a pendulum impact energy of 4 J and span of 60 mm. For the determination of the tensile strength and three-point bending strength, five specimens were tested, while for the determination of U-shaped notch impact strength, ten specimens were tested.

### 2.6. Micromorphology and Chemical Structure Characterization

Scanning electron microscopy (SEM) images of the surfaces of P/L, P/L-6, and P/L-8 before and after accelerated aging were obtained using a microscope (Quanta 200, FEI Company, Hillsboro, OR, USA) operated at 12.5 kV. To make the samples conductive, the sample surfaces were first sputtered with gold. Two specimens were used and six images were acquired for each specimen. Fourier transform infrared (FT-IR) spectra (ATR, Magna-IR560, Thermo Nicolet, Madison, WI, USA) of P/L, P/L-6, and P/L-8 before and after accelerated aging for 30 d were collected using a Nicolet Magna-IR 560 E.S.P. FT-IR spectrometer with the ATR technique in the range of 650–4000 cm^−1^. The spectra were recorded at a resolution of 4 cm^−1^ by accumulating 32 scans. Surface chemistry values for P/L, P/L-6, and P/L-8 before and after accelerated aging for 30 d were characterized via X-ray photoelectron spectroscopy (XPS) using a VG Thermo probe ESCA system (Thermo Electron, Palo Alto, CA, USA). The chemical shifts for C2, C3, and C4 relative to C1 (284.3 eV) were 1.5 ± 0.1 eV, 3.0 ± 0.1 eV, and 4.0 ± 0.1 eV.

## 3. Results and Discussion

### 3.1. Color Changes

**Figure 3:**
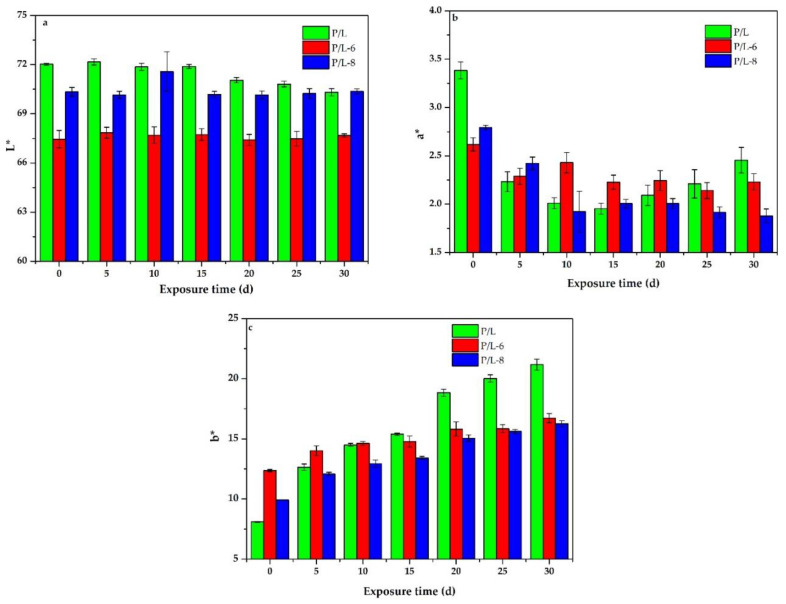
L*, a*, and b* values of samples before and after accelerated aging: (**a**) L*; (**b**) a*; and (**c**) b*.

Figure 3 displays the average L*, a*, and b* values for P/L, P/L-6, and P/L-8 before and after accelerated aging, while Table 2 provides the real color of samples and color differences of samples before and after accelerated aging. Notably, in Figure 3 and Table 2, the L* coordinate presents an overall slight descent trend, indicating that the accelerated aging caused a darkening of P/L. With increasing accelerated aging time, the L* coordinate barely changed first and declined rapidly afterwards. Specifically, the L* of P/L remained almost unchanged when accelerated aging was within 15 d and declined rapidly when accelerated aging was beyond 15 d. For P/L-6 and P/L-8, the L* coordinates varied little under accelerated aging. Arguably, the brightness (L* coordinate) levels of P/L-6 and P/L-8 were scarcely influenced by accelerated aging, implying that the addition of UV-326 and UV-328 into SLS parts significantly improved their brightness stability under the sun and rain.

The variations of the a* coordinate represent the change in the redness (+) and greenness (−). In other words, when the Δa* is positive, the redness increases; otherwise, the greenness improves. As shown in Figure 3 and Table 2, the a* coordinate of P/L decreased under accelerated aging, indicating that the greenness improved when the SLS parts were exposed to sun and rain. The decline occurred when the accelerated aging was within 5 d and the range of change of the a* coordinate was small after accelerated aging went beyond 5 d. Specifically, the decrease in the a* coordinate happened almost exclusively in the early stage of the accelerated aging. The a* coordinate change trend for P/L-6 was similar to that of P/L, which decreased rapidly in the early accelerated aging time of 5 d and remained almost constant in the later stage of accelerated aging. Nevertheless, the decline in the a* coordinate of P/L-6 was significantly less than that of P/L, suggesting that the improvement of greenness of SLS parts can be attributed to UV-326. The a* coordinate of P/L-8 also declined under accelerated aging, which is similar to change in P/L-6, with the only difference being that the a* coordinate of P/L-8 decreased rapidly in the first 10 d of accelerated aging and then remained almost constant after 10 d of accelerated aging. According to comprehensive comparisons, the reduction levels of the a* coordinates of SLS parts were as follows: P/L > P/L-8 > P/L-6. Therefore, it can be concluded that UV-326 and UV-328 had positive effects on preventing the color of the SLS parts from turning to green, and the effect of UV-326 was better than UV-328. 

The variations of the b* coordinate represent the change in the yellowness (+) and blueness (−). The yellowness improves when Δb* is positive; otherwise, the blueness improves. The b* coordinate of P/L improved rapidly with an increase in accelerated aging time, implying a rise of yellowness under accelerated aging. There were also improvements in the b* coordinates of P/L-6 and P/L-8 with increasing accelerated time, but the growth was slower than that of P/L, indicating that the addition of UV-326 or UV-328 can be advantageous for preventing a rise in the yellowness of SLS parts. The increase in the b* coordinate of P/L-6 was lower than that of P/L-8. Therefore, the UV-326 was more effective than UV-328 in preventing the SLS parts from turning to yellow.

As presented in Table 2, the trends of an overall color change for the ΔE* of SLS parts was similar to Δb*. ΔE* primarily depends on Δb*, which can be observed in the numerical analysis of ΔL*, Δa*, Δb*, and ΔE*. Hence, the overall color change ΔE* can be limited through dampening the growth of the b* coordinate, which can be achieved with UV-326 and UV-328. In conclusion, the overall color changes ΔE* of SLS parts can be effectively restrained by inhibiting the parts from turning to yellow via the addition of UV-326 and UV-328, while the effect of UV-326 was slightly stronger than UV-328.

### 3.2. Mechanical Properties

**Figure 4:**
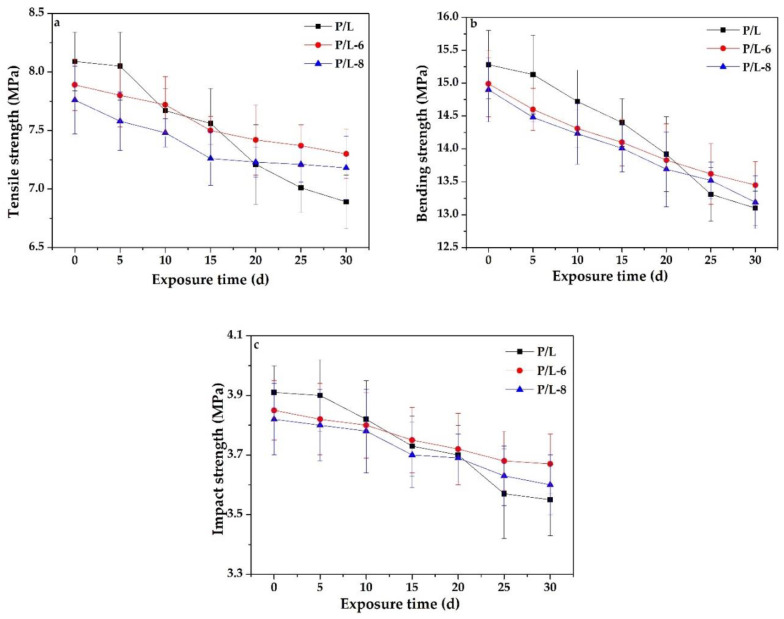
Mechanical properties of samples before and after accelerated aging: (**a**) tensile strength; (**b**) bending strength; and (**c**) impact strength.

The tensile strength, bending strength, and impact strength values of P/L, P/L-6, and P/L-8 before and after accelerated aging are shown in Figure 4. Before accelerated aging, the tensile strength, bending strength, and impact strength values of P/L were all higher than those of P/L-6 and P/L-8, indicating that the addition of UV-326 and UV-328 into the SLS parts was not beneficial for their mechanical properties. The tensile strength, bending strength, and impact strength values of all the specimens displayed a tendency to decrease with accelerated aging time, while those of the specimens with added UV-326 or UV-328 were higher than P/L after accelerated aging for 25 d. These results indicate that the SLS parts would suffer deterioration of the mechanical properties under the sun and rain, and that UV-326 and UV-328 can effectively protect them from weathering [9]. The mechanical properties of P/L-6 were higher than those of P/L-8, regardless of the accelerated aging time, implying that the addition of UV-326 had a more minor negative effect on the mechanical properties of SLS parts than UV-328 before accelerated aging, while it can also provide stronger protection to SLS parts against accelerated aging than UV-328. Therefore, UV-326 was slightly more effective than UV-328 in protecting the tensile strength, bending strength, and impact strength values of the SLS parts under accelerated aging.

### 3.3. Micromorphology

**Figure 5:**
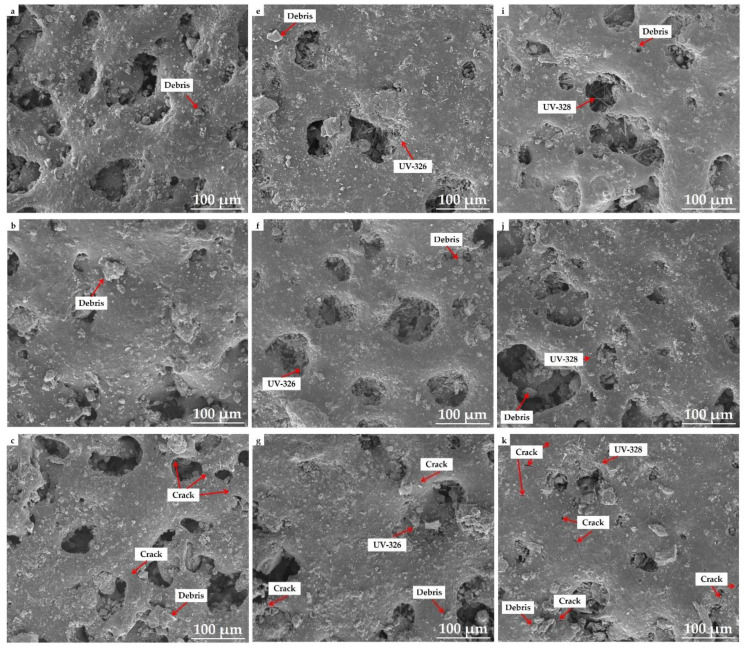

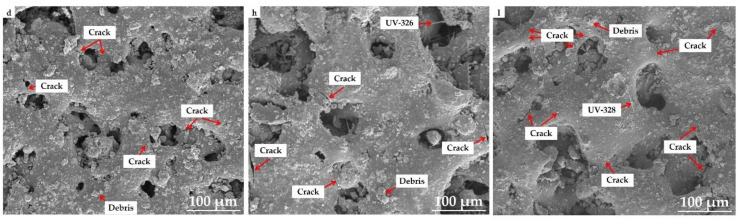
Micromorphologies of samples before and after accelerated aging: (**a–d**) P/L with accelerated aging for 0, 10, 20, and 30 d; (**e**–**h**) P/L-6 with accelerated aging for 0, 10, 20, and 30 d; and (**i**–**l**) P/L-8 with accelerated aging for 0, 10, 20, and 30 d.

Figure 5 shows the surface micromorphologies of P/L, P/L-6, and P/L-8 before and after accelerated aging. The initial surface of P/L was extremely coarse, with significant debris and several holes (Figure 5a). After accelerated aging for 10 d (Figure 5b), the surface was observed to be a little smoother with less debris, which was due to UV exposure and water washing for 10 days, which degraded the fragments and washed them off from the surface. With increasing accelerated aging from 10 d to 20 d (Figure 5c), the surface became a bit rougher with more detritus. Additionally, some cracks appeared on the surface after accelerated aging for 20 d, which could not be observed before. With further increase in accelerated aging up to 30 d (Figure 5d), the surface seemed to become much coarser, with many more fragments and cracks on the surface and in the holes. Hence, to conclude, the surface of P/L experienced severe damage after accelerated aging for 30 d, resulting in a decline in the mechanical performance and color fading.

Similar to P/L, the initial surface of P/L-6 was rough, with some debris and holes (Figure 5e). The needle-like objects on the surface and in the holes were UV-326 [10]. For P/L-6 that was subjected to accelerated aging for 10 d (Figure 5f), the surface became a little smoother and the debris decreased, which is because the surface was degraded and washed off under UV exposure and water rinsing. UV-326 was also degraded by UV exposure, which is reflected by the reduction in needle-like objects. With the increase in accelerated aging to 20 d (Figure 5g), novel cracks appeared on the surface and the surface became rougher, with a further reduction of detritus and UV-326. The cracks widened and increased with the increase in accelerated aging to 30 d (Figure 5h). Moreover, the surface continued coarsening, with more fragments and less UV-326.

As shown in Figure 5i–l, with increase in accelerated aging from 0 to 30 d, the change in the surface micromorphology for P/L-8 was practically identical to that of P/L-6. The only difference was that after 10 d of accelerated aging, the surface of P/L-6 became smoother with less debris due to degradation and flushing under UV exposure and water washing, while the surface of P/L-8 became rougher with more fragments. The reason for the phenomenon could be that the initial surface of P/L-8 was smoother with less debris than that of initial P/L-6, resulting in fewer fragments being scoured off from the surface of initial P/L-8 and the smooth surface being degraded into fragments, hence becoming rough after 10 d of accelerated aging. 

Comparing the surface micromorphology of P/L with those of P/L-6 and P/L-8, although the numbers of cracks in the SLS parts after accelerated aging for 30 d were similar, the surfaces of P/L-6 and P/L-8 after accelerated aging for 30 d were smoother than that of P/L. The UV-326 in P/L-6 and UV-328 in P/L-8 inhibited the degradation of SLS parts due to UV exposure and water washing via absorption of UV light, which resulted in their self-degradation and is reflected by the decreased amounts of UV-326 in P/L-6 and UV-328 in P/L-8. The degradation of SLS parts was inhibited, and thus fewer fragments were degraded from the surface, resulting in smoother surfaces for P/L-6 and P/L-8 than that of P/L after accelerated aging for 30 d. Therefore, the inhibition of degradation of SLS parts by UV-326 and UV-328 suppressed the decline in the mechanical properties of SLS parts.

### 3.4. FT-IR Analysis

**Figure 6:**
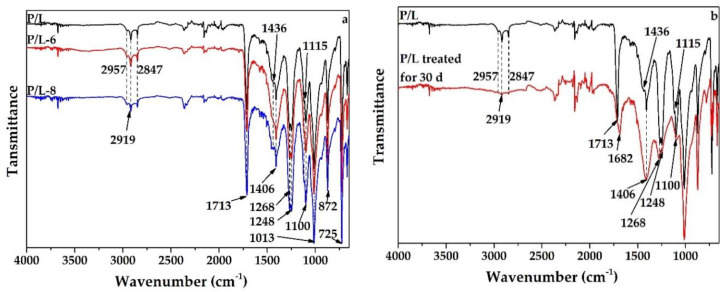

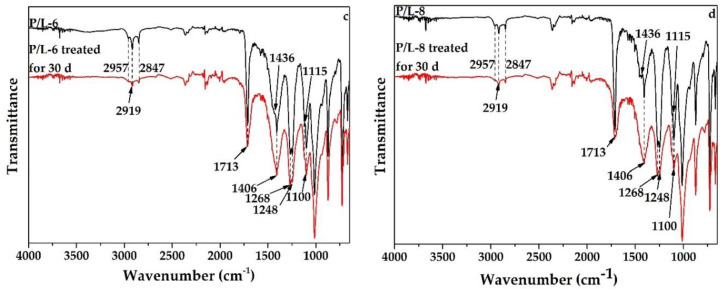
FT-IR spectra of samples before and after accelerated aging: (**a**) P/L, P/L-6, and P/L-8; (**b**) P/L before and after accelerated aging for 30 d; (**c**) P/L-6 before and after accelerated aging for 30 d; and (**d**) P/L-8 before and after accelerated aging for 30 d.

The FT-IR spectra of P/L, P/L-6, and P/L-8 before and after accelerated aging for 30 d are shown in Figure 6. The analysis was performed to identify the key functional groups responsible for the improvement in weather resistance of SLS parts due to UV-326 and UV-328. In the analysis of the initial spectra of P/L, P/L-6, and P/L-8 (Figure 6a), the main peaks for copolyester were assigned as follows: two minor peaks at 2957 and 2919 cm^−1^ corresponded to the asymmetric stretching vibrations of CH groups [11,12], a peak at 2847 cm^−1^ corresponded to the symmetric stretching vibrations of CH groups [13], a peak at 1713 cm^−1^ was attributed to the stretching of carbonyl groups (C=O) present in ester groups [14], a peak at 1406 cm^−1^ corresponded to OH bending [15], two peaks at 1268 and 1248 cm^−1^ corresponded to C-O-C asymmetric stretching in ester group [16], two peaks at 1115 and 1100 cm^−1^ were due to C-O stretching in the ester group [17], and a peak at 1013 cm^−1^ could be attributed to C-O stretching in hydroxyl groups [18]. The following peaks were assigned for limestone: a peak at 1436 cm^−1^ corresponded to asymmetric C-O stretching vibration, a peak at 872 cm^−1^ could be attributed to symmetric C-O stretching vibration, and a peak at 725 cm^−1^ was due to OCO bending (in-plane deformation) vibration [19]. There were only minor differences in the initial spectra of P/L, P/L-6, and P/L-8, indicating that the small amount of UV-326 or UV-328 in P/L had nearly no effects on its chemical structure. 

The FT-IR spectra of P/L after accelerated aging for 30 d was similar to that of the initial P/L, with minor shifts and changes in peak intensities corresponding to the effect of accelerated aging on the chemical structure of P/L (Figure 6b). The significant decreases in intensities of the peaks at 2957 cm^−1^, 2919 cm^−1^, and 2857 cm^−1^ were due to the reduction of CH groups due to UV exposure and water washing. The peak at 1713 cm^−1^ was shifted to 1682 cm^−1^ and broadened, indicating that some ester groups (O-C=O) turned into carbonyl groups (C=O) and carboxyl groups (COOH). The widening of the peak at 1406 cm^−1^ was due to the increase in the OH groups. The slump in intensities of peaks at 1268 and 1248 cm^−1^ indicated that a large number of ester groups degraded under UV exposure and water washing. The disappearance of the peak at 1115 cm^−1^ present in the initial P/L and a clear weakening in the intensity of the peak at 1100 cm^−1^ were also due to the degradation of a large number of ester groups. The above results signify that CH and ester groups in copolyester were degraded and hydroxyl, carbonyl, and carboxyl groups were generated under UV exposure and water washing. The peak at 1436 cm^−1^ corresponding to the asymmetric C-O stretching vibration in CaCO_3_ present in the initial P/L merged with the peak at 1406 cm^−1^, while there were almost no changes in the intensities and wavenumbers of peaks at 872 and 725 cm^−1^, implying that there was only a slight effect of accelerated aging on the chemical structure of CaCO_3_.

In contrast, as per the analysis of spectra of P/L-6 and P/L-8 before and after accelerated aging for 30 d (Figure 6c,d), the changes in intensities and wavenumbers of the peaks at 2957, 2919, 2857, 1713, 1406, 1268, 1248, 1115, and 1100 cm^−1^ in the spectra of P/L-6 and P/L-8 after accelerated aging for 30 d were weaker than those of P/L. This phenomenon implies that fewer CH groups and ester groups were degraded, and hence fewer hydroxyl, carbonyl, and carboxyl groups were generated under UV exposure and water washing due to the addition of UV-326 and UV-328, which effectively protected the SLS parts. 

### 3.5. XPS Analysis

**Figure 7:**
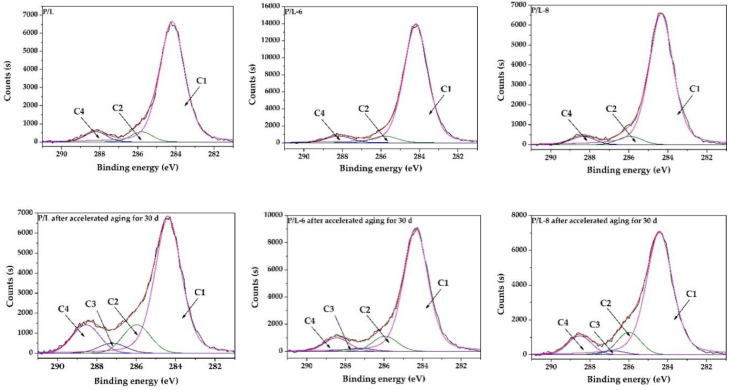
High-resolution XPS spectra of samples before and after accelerated aging.

The XPS analysis was employed to identify the surface chemistry of SLS parts. Figure 7 shows the C1s spectra with high-resolution scans for P/L, P/L-6, and P/L-8 before and after accelerated aging for 30 d. The spectra were deconvoluted into four components for all of the specimens: C1 (C-C, C-H), C2 (C-O), C3 (C=O/O-C-O), and C4 (O-C=O) [20]. Compared to the initial P/L, the C/1 of P/L after accelerated aging for 30 d decreased a lot, while C2, C3, C4, and the O/C ratio increased (Table 3). Combined with the results of the FT-IR analysis, the decrease in C1 and increase in C2, C3, C4, and O/C ratio after accelerated aging for 30 d can be attributed to the degradation of CH groups and ester groups and the generation of hydroxyl, carbonyl, and carboxyl groups. The C1 of the initial P/L-6 and P/L-8 samples increased a little as compared to the initial P/L sample, while C2, C4, and the O/C ratio decreased slightly due to the addition of UV-326 and UV-328. After accelerated aging for 30 d, the C1 levels of P/L-6 and P/L-8 decreased, and C2, C3, C4, and O/C ratio increased. This phenomenon indicates that similar changes happened on P/L-6 and P/L-8 under UV exposure and water washing. However, after accelerated aging for 30 d, the C1 levels of P/L-6 and P/L-8 were greater than that of P/L, while the C2, C3, and C4 levels and the O/C ratio were less than those of P/L. These results imply that the addition of UV-326 and UV-328 into SLS parts inhibited the degradation of CH and ester groups and the generation of hydroxyl, carbonyl, and carboxyl groups under UV exposure and water washing.

In summary, as per the results of the color changes, the mechanical properties, the micromorphologies, the FT-IR analysis, and the XPS analysis, the modification of SLS parts under UV exposure and water washing and the improvement mechanism of the weather resistance of SLS parts due to UV-326 and UV-328 can be suggested as the underlying causes. Under accelerated aging, the CH and ester groups of P/L suffered degradation, and thus the hydroxyl, carbonyl, and carboxyl groups were generated. With the degradation of CH and ester groups and the formation of oxygen-containing groups, the surface of P/L slowly cracked and the bulk material of P/L gradually degraded into small fragments, resulting in a decline of the mechanical properties. Moreover, changes in the chemical structure of P/L under accelerated aging led to an increase in yellowness. Finally, P/L suffered a decline of its mechanical properties and turned yellow due to sun and rain conditions. The addition of UV-326 or UV-328 inhibited the degradation of CH and ester groups and the generation of hydroxyl, carbonyl, and carboxyl groups, and thus restrained the deterioration of mechanical properties and yellowing of SLS parts. The inhibition of the deterioration of mechanical properties and yellowing of SLS parts due to UV-326 was stronger than the effect of UV-328; however, the distinction was not significant.

## 4. Conclusions

To address the issue of deterioration of the color and shape of precious, ancient structures built with stone and brick caused by the sun and rain, limestone powder and copolyester were employed as raw materials to prepare parts for repair of these buildings using SLS technology. To improve the weather resistance of these SLS parts, UV-326 and UV-328 were added and the improvement of weather resistance was examined via accelerated aging tests. The addition of UV-326 and UV-328 effectively inhibited the yellowing and deterioration of the mechanical properties of SLS parts under sun and rain conditions by restraining the degradation of CH and ester groups and the generation of hydroxyl, carbonyl, and carboxyl groups. Owing to the significant enhancement of the weather resistance, SLS parts with UV-326 or UV-328 as a UV stabilizer have great potential for the repair and maintenance of ancient, precious brick buildings.

## Figures and Tables

**Table 1:** Main parameters of the selective laser sintering (SLS) process.

Laser Power	Scanning Speed	Layer Thickness	Scan Spacing	Processing Temperature
30 W	2000 mm/s	0.2 mm	0.15 mm	175 °C

**Table 2:** Colors of samples before and after accelerated aging.

		Accelerated Aging Time (d)						
		0	5	10	15	20	25	30
P/L	Color	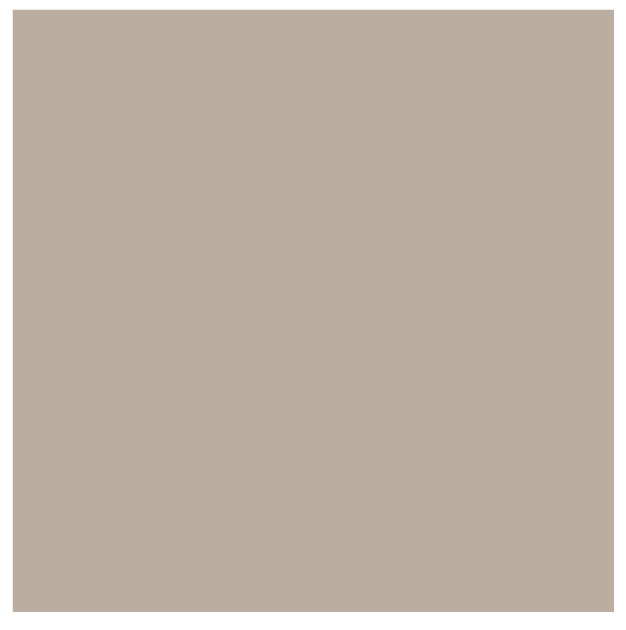	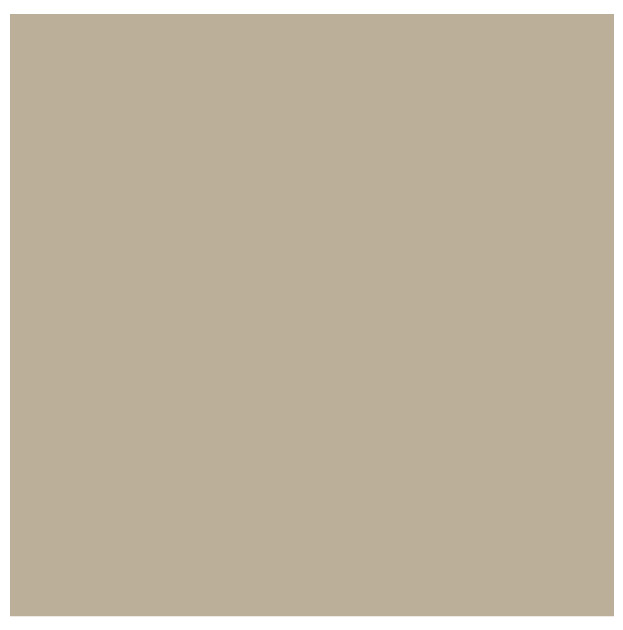	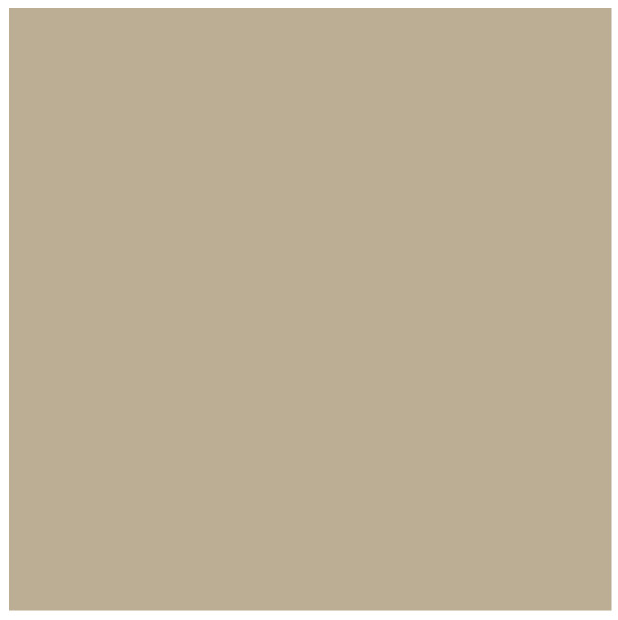	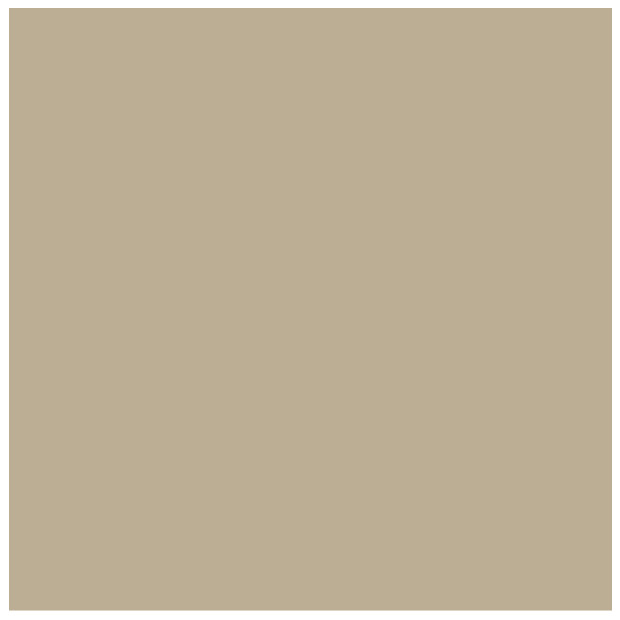	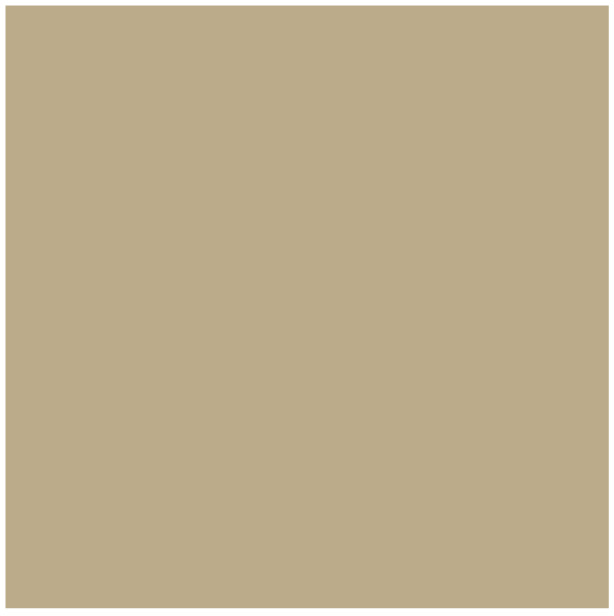	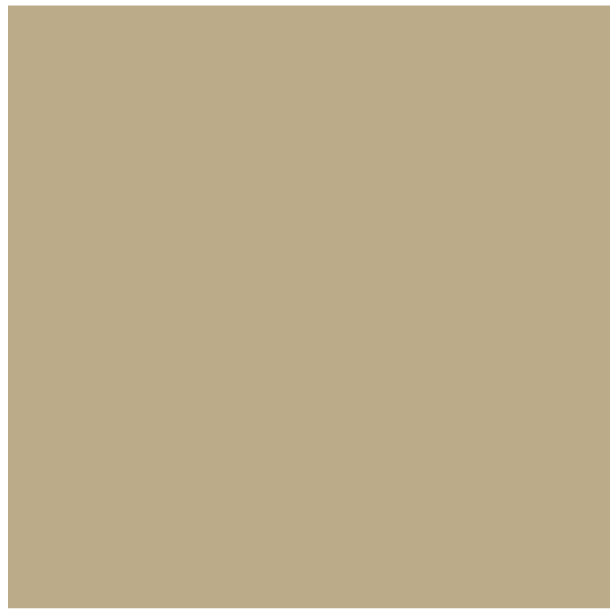	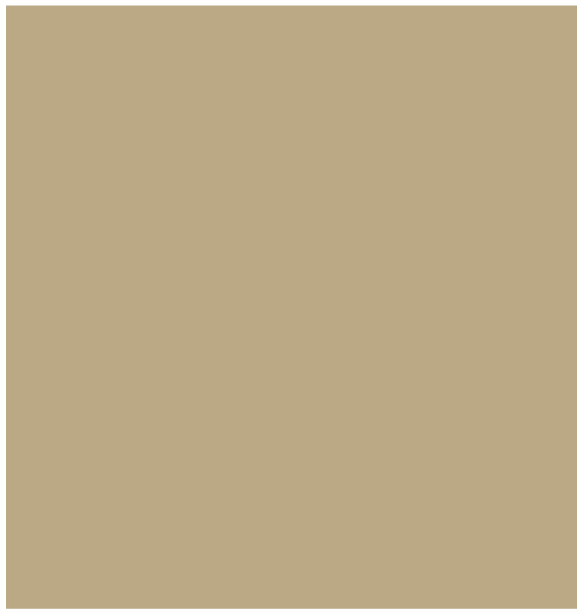
	ΔL*	-	0.14	−0.16	−0.14	−0.97	−1.21	−1.72
	Δa*	-	−1.15	−1.37	−1.43	−1.29	−1.17	−0.93
	Δb*	-	4.55	6.41	7.29	10.75	11.93	13.08
	ΔE*	-	4.7	6.56	7.43	10.87	12.05	13.23
P/L-6	Color	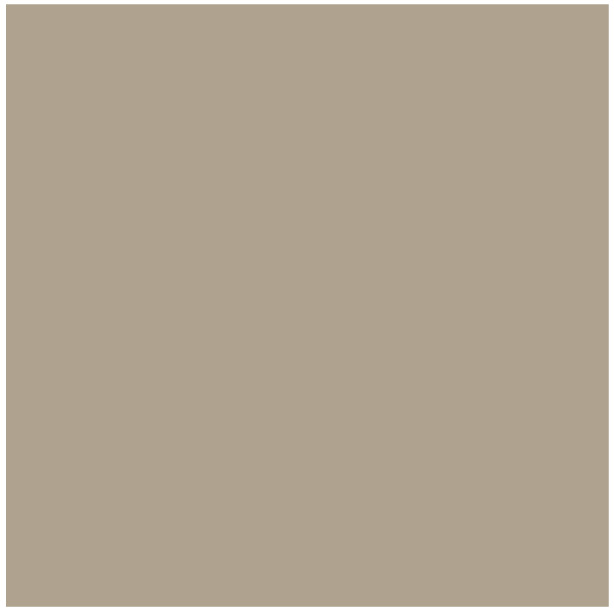	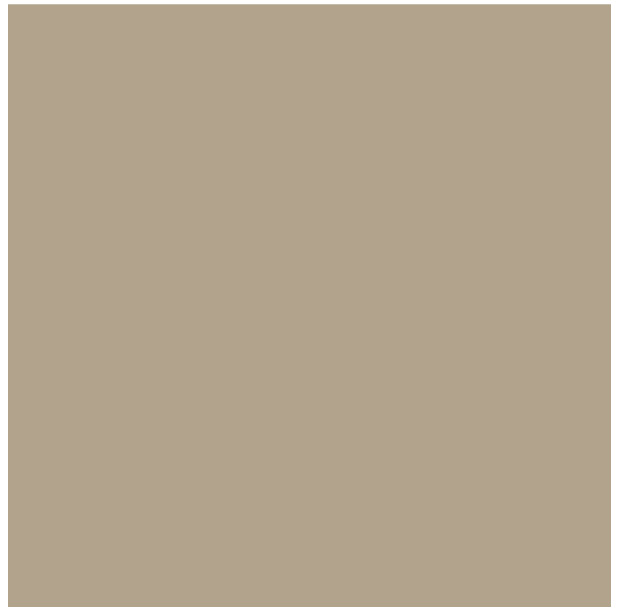	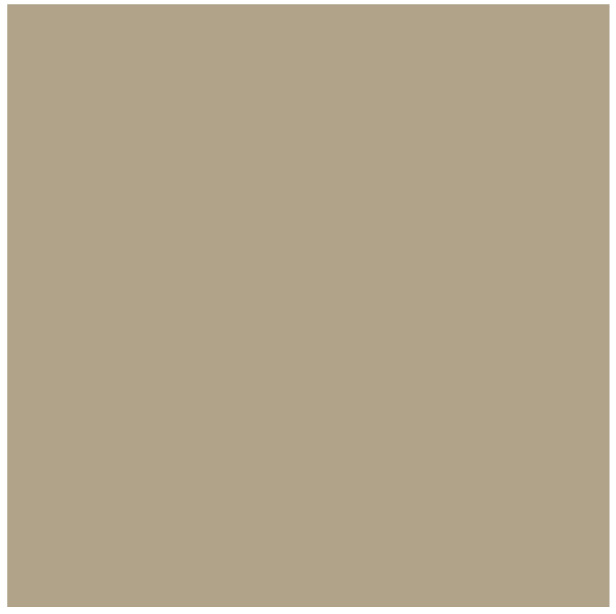	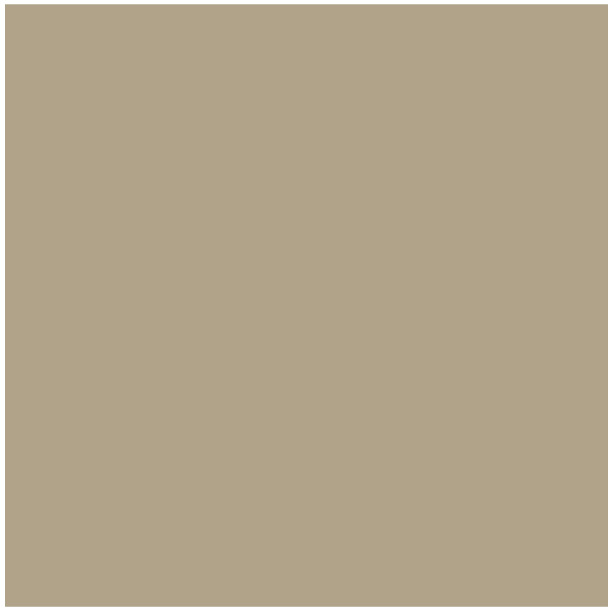	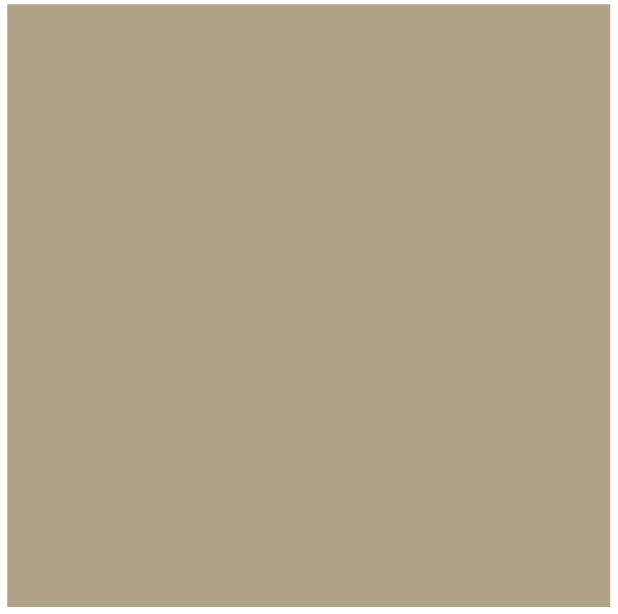	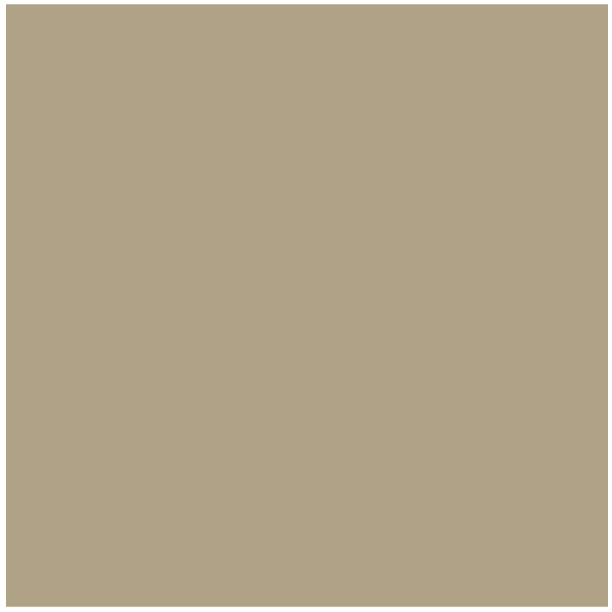	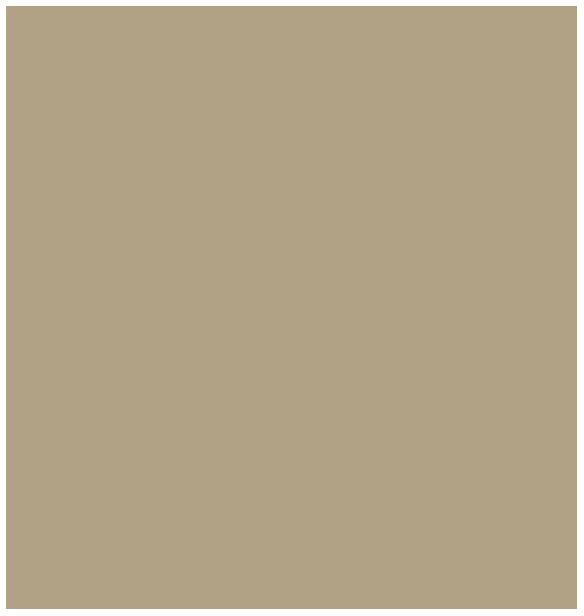
	ΔL*	-	0.4	0.25	0.28	−0.04	0.03	0.23
	Δa*	-	−0.33	−0.19	−0.39	−0.37	−0.48	−0.39
	Δb*	-	1.65	2.27	2.41	3.46	3.48	4.36
	ΔE*	-	1.73	2.29	2.46	3.48	3.51	4.39
P/L-8	Color	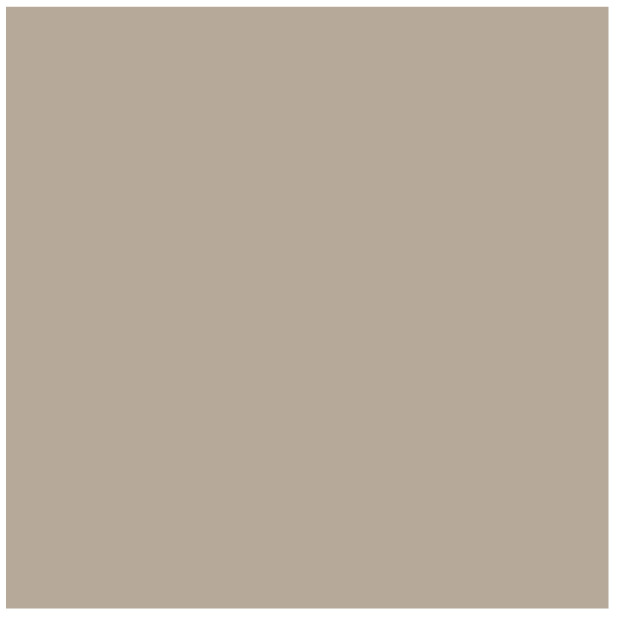	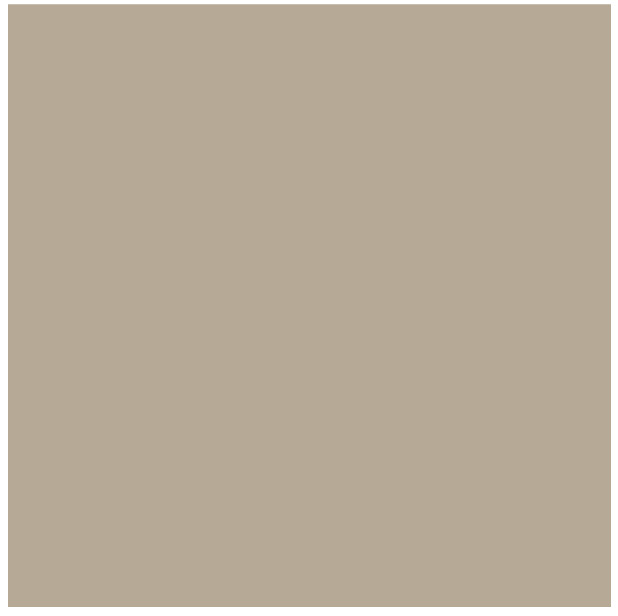	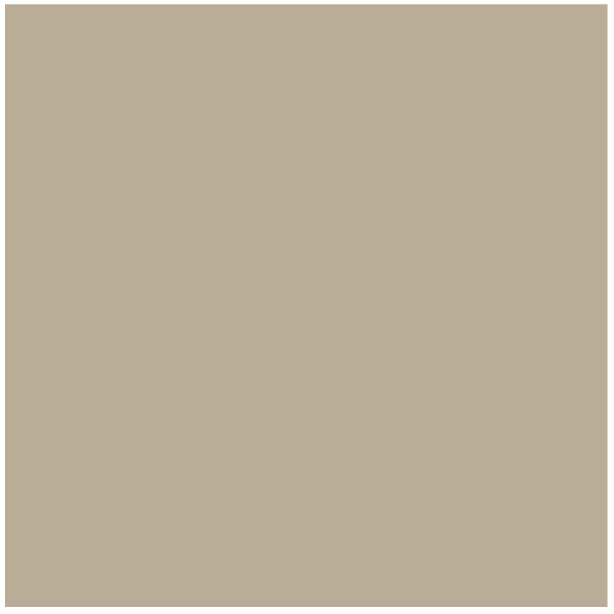	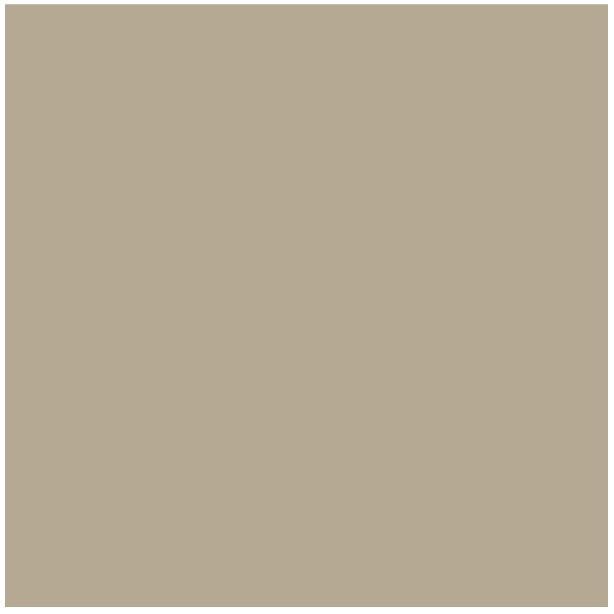	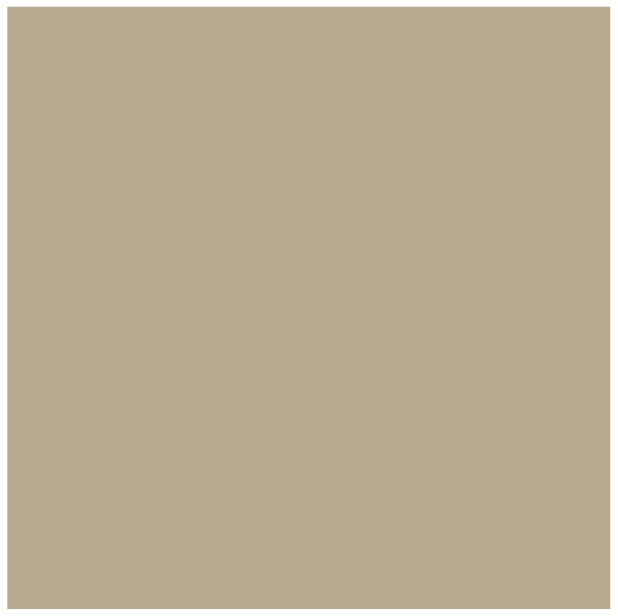	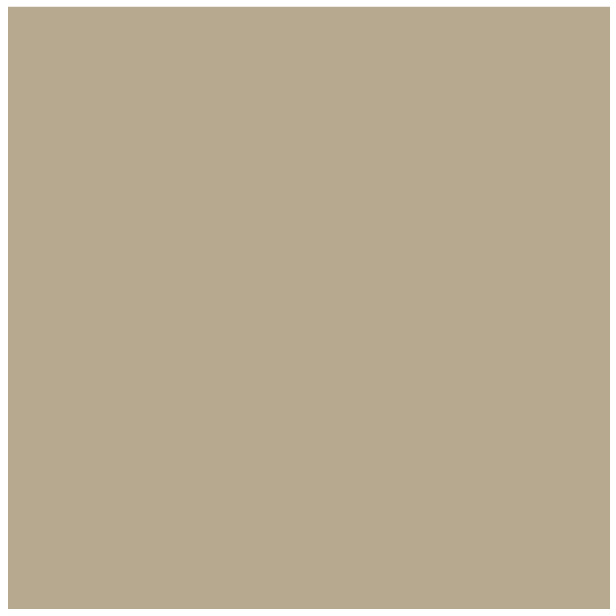	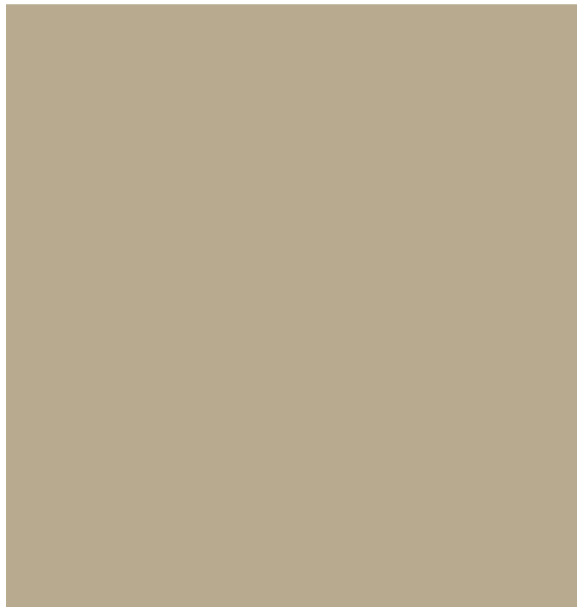
	ΔL*	-	−0.19	1.24	−0.16	−0.19	−0.11	0.02
	Δa*	-	−0.37	−0.87	−0.79	−0.79	−0.88	−0.91
	Δb*	-	2.16	3.02	3.5	5.14	5.7	6.35
	ΔE*	-	2.2	3.38	3.59	5.21	5.77	6.42

**Table 3:** Surface chemistry components of SLS parts as determined by XPS.

Sample	Elements (%)		Carbon Species C1s (%)				O/C Ratio
	C	O	C1	C2	C3	C4	
P/L	75.2	24.8	88.7	5.6	0	5.7	0.33
P/L after accelerated aging for 30 d	64.8	35.2	69.2	13.1	4.6	13.1	0.54
P/L-6	76.9	23.1	90.1	4.9	0	5.0	0.30
P/L-6 after accelerated aging for 30 d	69.4	30.6	81.8	8.7	1.8	7.7	0.44
P/L-8	80.5	19.5	90.6	4.7	0	4.7	0.24
P/L-8 after accelerated aging for 30 d	70.6	29.4	78.1	10.9	1.9	9.1	0.42
